# Radical Scavenging Mechanisms of Phenolic Compounds: A Quantitative Structure-Property Relationship (QSPR) Study

**DOI:** 10.3389/fnut.2022.882458

**Published:** 2022-04-04

**Authors:** Melanie Platzer, Sandra Kiese, Thorsten Tybussek, Thomas Herfellner, Franziska Schneider, Ute Schweiggert-Weisz, Peter Eisner

**Affiliations:** ^1^TUM School of Life Sciences Weihenstephan, ZIEL-Institute for Food & Health, Technical University of Munich, Freising, Germany; ^2^Fraunhofer Institute for Process Engineering and Packaging IVV, Freising, Germany; ^3^Chair of Food Science, Institute for Nutritional and Food Sciences, University of Bonn, Bonn, Germany; ^4^Faculty of Technology and Engineering, Steinbeis-Hochschule, Dresden, Germany

**Keywords:** area under the curve, antioxidant effect, flavonoids, phenolic acids, structure-activity relationship

## Abstract

Due to their antioxidant properties, secondary plant metabolites can scavenge free radicals such as reactive oxygen species and protect foods from oxidation processes. Our aim was to study structural influences, like basic structure, number of hydroxyl groups and number of Bors criteria on the outcome of the oxygen radical absorbance capacity (ORAC) assay. Furthermore, similarities and differences to other *in vitro* antioxidant assays were analyzed by principal component analysis. Our studies confirmed that the antioxidant behavior in the ORAC assay is dominated by the number and types of substituents and not by the Bors criteria, as long as no steric hindrance occurs. For example, morin (MOR) with five hydroxyl groups and two Bors criteria reached an area under the curve of (3.64 ± 0.08) × 10^5^, which was significantly higher than quercetin-7-D-glucoside (QGU7) (*P* < 0.001), and thus the highest result. Principal component analysis showed different dependencies regarding structural properties of Folin-Ciocalteu (FC)- and 2,2-diphenyl-1-picrylhydrazyl (DPPH)-assays or 2,2'-azino-bis (3-ethylbenzothiazoline-6-sulfonic acid) (ABTS)- and ORAC-assays, respectively. Therefore, we conclude that they are based on different reaction mechanisms. The number of hydroxyl groups showed a stronger influence on the antioxidant activity than the Bors criteria. Due to these differences, the correlation of these rapid tests to specific applications should be validated.

## 1. Introduction

**Figure 1:**
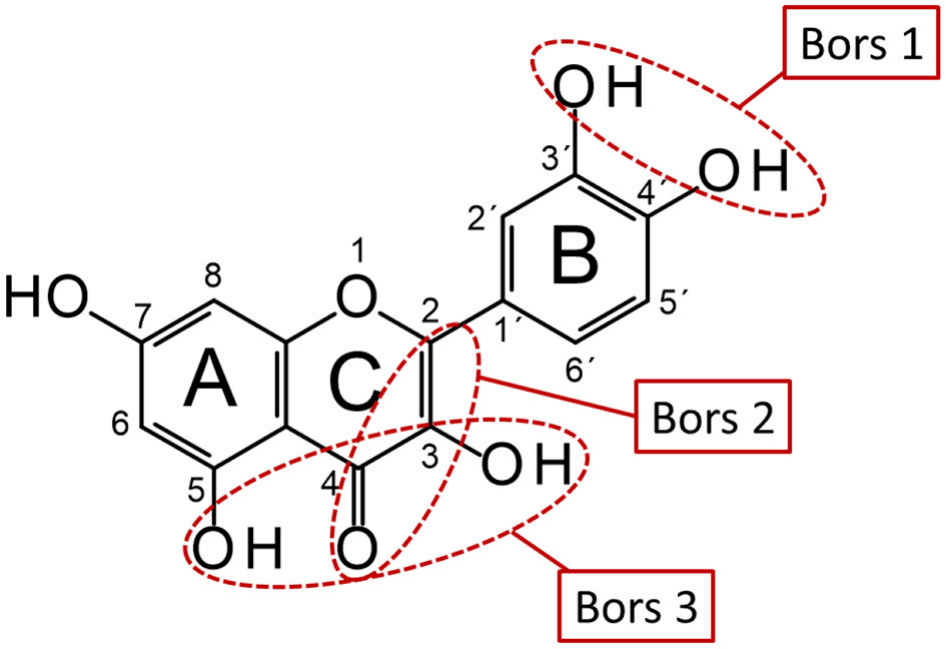
Schematic representation of Bors criteria using QUR. Bors 1: catechol group on the B-ring; Bors 2: double bond between C-2 and C-3 and a carbonyl group at C-4 on the C-ring; Bors 3: hydroxyl groups at C-3 and C-5 on the A- and C-rings and 4-oxo group on the C-ring (reproduced from Platzer et al. (11)).

Secondary plant metabolites are responsible for various functions in plants, such as protection against herbivores, UV radiation, pests and pathogens (1–3). Since these compounds are found more abundantly in the edible parts of plants and are also known for their health-promoting and disease-preventing properties, they are of particular interest to the food and pharmaceutical industries. Due to their antioxidant properties, they can scavenge free radicals such as reactive oxygen species and protect foods from oxidation processes, significantly improving their storage stability and quality (1, 4–8). Phenolic compounds, which are prominent representatives of secondary plant metabolites, are among the most important naturally occurring antioxidants and can be classified into different subgroups based on their structural properties. In addition to low-molecular compounds such as phenolic acids, there are several, more complex representatives including the group of flavonoids (9, 10). The strength of the antioxidant effect of these compounds has been described in numerous publications and depends on their structural properties, such as the Bors criteria (Figure 1) (11–14).

Analytical methods, such as the 2,2'-azino-bis (3-ethylbenzothiazoline-6-sulfonic acid) (ABTS), 2,2-diphenyl-1-picrylhydrazyl (DPPH), oxygen radical absorbance capacity (ORAC) or Folin-Ciocalteu (FC) assays are often used to measure the antioxidant activity (15–18). These rapid tests can be distinguished based on the underlying reaction mechanism, the single electron transfer (SET) and the hydrogen atom transfer (HAT), which run both in parallel, but at different rates (19–24).

SET-based assays, such as ABTS, DPPH, and FC (18, 19, 25–27), measure the electron loss of a free radical (R^·^) resulting in a radical anion and the reaction mechanisms were described elsewhere (11, 14). In the HAT-based mechanism (Reaction 1) a hydrogen atom is delivered from the antioxidant (AOH) directly to a radical to interrupt the oxidative chain reaction.


(1) $$\begin{array}{r} R^{\cdot }+AOH->RH+AO^{\cdot } \end{array}$$

The ORAC assay is a popular example of a HAT-based assay and is particularly used in the food industry (18, 19, 25–27). In contrast to the ABTS, DPPH, and FC assays, the measurement principle is not based on a color loss or color change, but on a decrease in fluorescence (19, 28–30). The presence of antioxidants neutralizes the peroxyl radicals by either a hydrogen atom transfer or a radical addition, which results in the fluorescence decrease being slowed down (30, 31). This decrease is measured as a function of time and evaluated subsequently as AUC (AUC) (30, 31). The AUC represents the antioxidant effect of a substance and, in contrast to other evaluation methods, combines the inhibition time and the amount of free radicals neutralized by antioxidants (32–34). Therefore, the test is suitable for antioxidants with or without a pronounced lag phase, which is particularly useful for food samples that often consist of several different ingredients (30, 35). Furthermore, the assay is suitable for both hydrophobic and hydrophilic substances (31). Although the ORAC assay is a widely used method, little is known about structural properties that have an impact on the measurement results. In the literature, there are mainly theoretical studies (20, 23, 24, 36–43) on the commonalities of the two reaction mechanisms and less systematic experimental studies on the correlations of different assays in terms of influence of the basic structure, number of hydroxyl groups and number of fulfilled Bors criteria. In addition, not much is reported on whether the same structural properties are crucial for SET- and HAT-based assays and how they differ from each other.

Therefore, we investigated the influence of structural properties of phenolic compounds on the outcome of the ORAC assay. For this purpose, different standard references belonging to the subgroups of phenolic acids, flavonols, flavanones, dihydrochalcones, and flavanols were analyzed. Furthermore, we compared these results to those from ABTS, DPPH and FC assays to investigate common trends and differences regarding structural properties leading to high antioxidant behavior. To investigate this in more detail, the results were clustered according to different criteria and presented in a principal component analysis. Special attention was paid to the subgroup, the number of hydroxyl groups, the number of fulfilled Bors criteria as well as the reaction mechanism in the respective assay.

## 2. Materials and Methods

Chemicals and reference standards (see Table 1 slightly modified from Table 1 in Platzer et al. (11, 14)) were obtained from Sigma-Aldrich (Steinheim, Germany): 2,2'-azubis-(2'-methylpropionamidine) dihydrochloride (AAPH), caffeic acid (CAA), (+)-catechin (CAT), 3,4-dihydroxybenzoic acid (DBA), (−)-epicatechin (EPC), ferulic acid (FEA), fluorescein, gallic acid (GAA), 4-hydroxybenzoic acid (HBA), hesperetin (HES), kaempferol (KAE), morin (MOR), myricetin (MYR), naringenin (NAN), *p*-coumaric acid (PCA), phloridzin (PHD), phloretin (PHT), quercetin-3-D-glucoside (QGU3), quercetin-7-glucoside (QGU7), quercetin (QUR), sinapic acid (SIA), syringic acid (SRA), taxifolin (TAF) and Folin-Ciocalteu (FC) reagent. The standard reference narirutin (NAR) was obtained from K&J Scientific (Marbach am Neckar, Germany) and isorhamnetin (IRT) and naringin (NAG) from Carl Roth (Karlsruhe, Germany). Stock solutions were prepared by dissolving the reference standards in analytical grade ethanol_absolute_ and diluting each of them in seven steps (2E-4 to 1E-1 mM) for the measurements.

**Table 1:** Phenolic compounds analyzed in this study (phenolic acids and subgroups of flavonoids) along with reference standard names, sample codes and substituents (reproduced and slightly modified from this table in Platzer et al. (11, 14)).

**Subgroups**	**Reference standards**	**Code**	**Position and substituents**						
**Phenolic acids**			**1**	**3**	**4**	**5**			
	Caffeic acid	CAA	(CH_2_)_2_COOH	OH	OH	H			
	3,4-dihydroxybenzoic acid	DBA	COOH	OH	OH	H			
	Ferulic acid	FEA	(CH_2_)_2_COOH	OCH_3_	OH	H			
	Gallic acid	GAA	COOH	OH	OH	OH			
	4-hydroxybencoic acid	HBA	COOH	H	OH	H			
	*p*-coumaric acid	PCA	(CH_2_)_2_COOH	H	OH	H			
	Sinapic acid	SIA	(CH_2_)_2_COOH	OCH_3_	OH	OCH_3_			
	Syringic acid	SRA	COOH	OCH_3_	OH	OCH_3_			
**Flavonols**			**2'**	**3'**	**4'**	**5'**	**3**	**5**	**7**
	Isorhamnetin	IRT	H	OCH_3_	OH	H	OH	OH	OH
	Kaempferol	KAE	H	H	OH	H	OH	OH	OH
	Morin	MOR	OH	H	OH	H	OH	OH	OH
	Myricetin	MYR	H	OH	OH	OH	OH	OH	OH
	Quercetin-3-D-glucoside	QGU3	H	OH	OH	H	Gal	OH	OH
	Quercetin-7-D-glucoside	QGU7	H	OH	OH	H	OH	OH	Glc
	Quercetin	QUR	H	OH	OH	H	OH	OH	OH
**Flavanones**			**3'**	**4'**	**3**	**5**	**7**		
	Hesperetin	HES	OH	OCH_3_	H	OH	OH		
	Narirutin	NAR	H	OH	H	OH	2 Glc		
	Naringin	NAG	H	OH	H	OH	Rham, Glc		
	Naringenin	NAN	H	OH	H	OH	OH		
	Taxifolin	TAF	OH	OH	OH	OH	OH		
**Dihydrochalcones**			**4**	**2'**	**4'**	**6'**			
	Phloridzin	PHD	OH	OH	OH	Glc			
	Phloretin	PHT	OH	OH	OH	OH			
**Flavanols**			**3'**	**4'**	**3**	**4**	**5**	**7**	
	(+)-catechin	CAT	OH	OH	OH	H	OH	OH	
	(−)-epicatechin	EPC	OH	OH	OH	H	OH	OH	

The AUC was determined using the ORAC assay according to the procedure of Huang et al. (44) with slight modifications. The stock solution of fluorescein was prepared by dissolving 1.33 mg in 100 ml 75 mM phosphate buffer and stored in the dark at 4 °C. The working solution, which was made freshly every day, was prepared by diluting the stock solution 1:1,000 with 75 mM phosphate buffer. For the AAPH solution, which was also freshly prepared daily, we dissolved 414 mg in 10 ml of the 75 mM phosphate buffer and stored the solution at 4°C until use. For the measurement, a 96-well plate was used, where the outer cells were filled with water. 25 μl of each sample together with 150μl of fluorescein stock solution were added to the wells and incubated for 30 min at 37°C in a microplate reader (Infinite 230 PRO, TECAN (Männedorf, Switzerland). The reaction was started by addition of 25 μl AAPH solution and fluorescence was recorded every 60 s, after shaking the plate for 10 s, as kinetics over time (excitation wavelength 485 nm, 20 nm bandpass, emission wavelength 535 nm, 20 nm bandpass). Each sample was measured in triplicate.

The raw data were evaluated following Cao and Prior (45) with some modifications. The obtained curves were normalized to their initial value and subsequently integrated to obtain the AUC. Net AUC (AUC_*sample*_ - AUC_*blank*_) was plotted as a function of AOH concentration. To calculate the value of a 1 mM solution, a linear regression equation was used. In order to not underestimate the AUC, only the concentrations below saturation were used as already described in Platzer et al. (14).

The results from the ABTS, DPPH and FC assays are shown here for comparison only and do not represent new results. Therefore, the corresponding method descriptions can be found in the literature and are not repeated here (11, 14).

For statistical analysis, a one-way analysis of variance (ANOVA) was performed using Sigma Plot (Systat Software, San Jose, CA, USA), equivalent to an unpaired *t*-test. If a significant difference was found, an additional paired test using the Holm-Sídák method was added. The significance level for both tests was at *P* < 0.05. Statistical analysis was always performed with all significant decimal places. The performance of the statistical analyzes was carried out for all measured substances. To investigate significant differences within the phenolic subgroups, the statistical analysis was additionally performed within the respective group and reference was included at the corresponding positions in the text. A multivariate analysis was performed by principal component analysis using OriginPro2018.

## 3. Results and Discussion

Table 1 shows the phenolic compounds used in this study and their five different subgroups (slightly modified from Table 1 in Platzer et al. (11, 14)).

### 3.1. Consolidated Analysis of Phenolic Subgroups in the ORAC Assay

**Figure 2:**
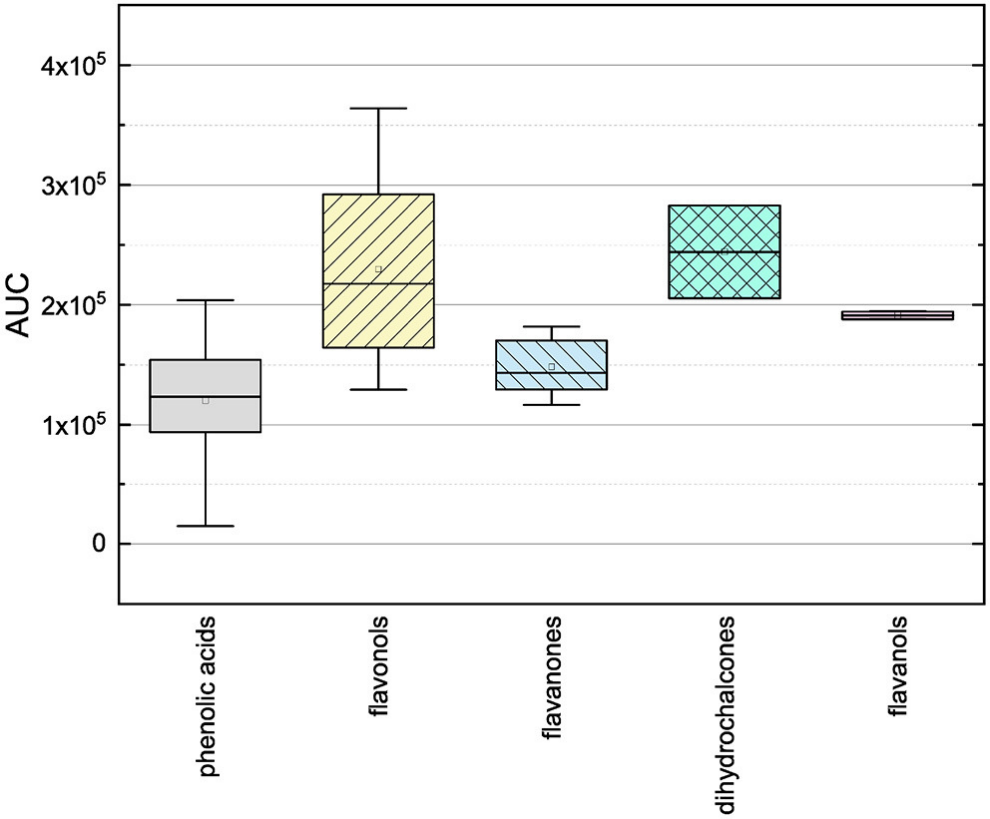
Boxplot of the mean values of AUC of different subgroups of phenolic compounds (phenolic acids, flavonoles, flavanones, dihydrochalcones, flavanoles) in the ORAC assay. The range within standard errors (1.5 interquartile range) is represented by error bars.

In order to investigate the antioxidant behavior of the phenolic subgroups, the results of each subgroup were averaged and are presented in Figure 2 as boxplot.

The phenolic acids, the flavanones and the flavanols obtained lower mean values and the flavonols and dyhydrochalcones slightly higher mean values. The low mean values of the phenolic acids and the flavanones could be explained by their low number of hydroxyl groups (phenolic acids 1–3, flavanones 2–5). The substances used, with exception of TAF, do not meet any Bors criteria. The flavanols also reached a low mean value, but it should be noted that only two substances were measured in this group. As those two substances have five hydroxyl groups and fulfill the first Bors criterion, a higher value was expected. The dihydrochalcones having three or four hydroxyl groups reached the second highest mean value. Here, a lower value was expected because the substances do not fulfill any of the Bors criteria and also have a lower number of hydroxyl groups than the flavanols. The flavonols reached the highest mean value, which could be explained by their high number of hydroxyl groups (4–6) and by the fact that most of the Bors criteria are fulfilled. Thus, the result in the ORAC assay seems to depend on both the number of hydroxyl groups and the fulfilled Bors criteria. In order to analyze this influence in more detail, the results were rearranged and are discussed later (see Section 3.3).

The mean values of the substances that do not fulfill any of the Bors criteria is not significantly different from those that fulfill one, two or three criteria. Considering the influence of the number of hydroxyl groups on the molecule, the mean value increased with increasing number of hydroxyl groups. However, substances with five hydroxyl groups partially showed a lower antioxidant behavior, with the exception of MOR, which also explains the outlier in the graph. MYR, which has six hydroxyl groups showed a significantly lower value. From these results we assume that steric hindrance could lead to a reduction of the antioxidant potential.

To analyze the structural properties of the phenolic compounds in more detail, the individual results of each substance are discussed in the following.

### 3.2. Analysis of Individual Reference Standards in the ORAC Assay

**Figure 3:**
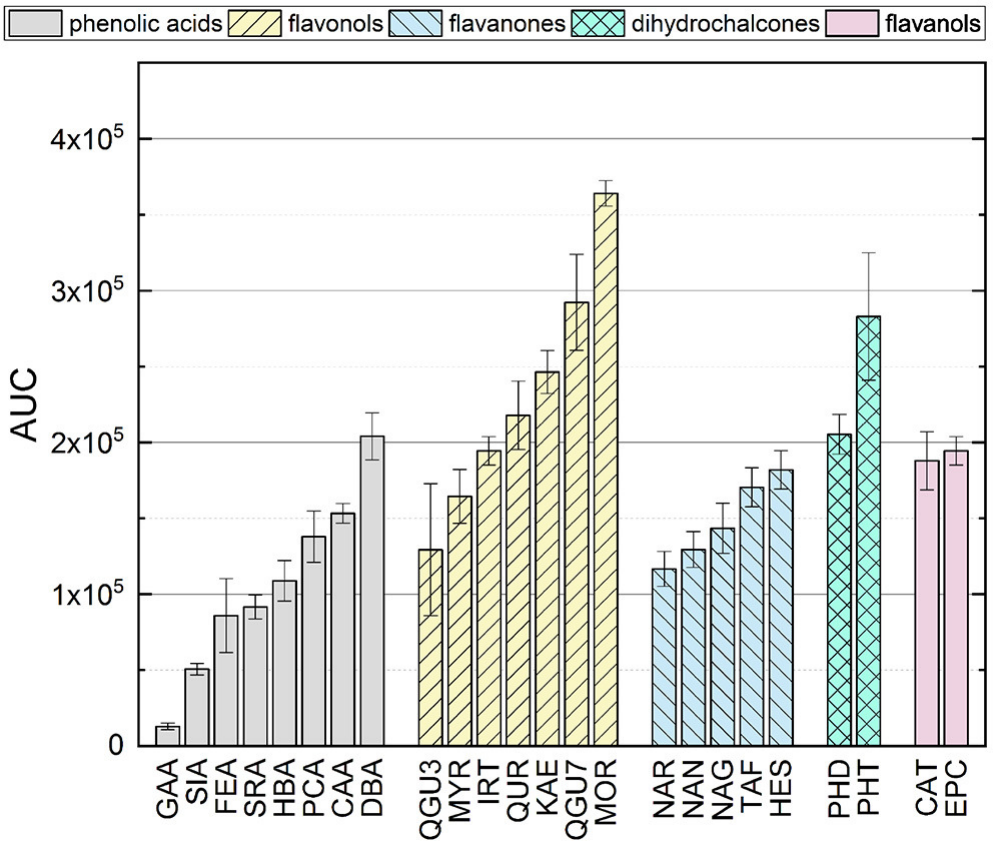
AUC of all standard reference compounds determined by ORAC assay.

**Figure 4:**
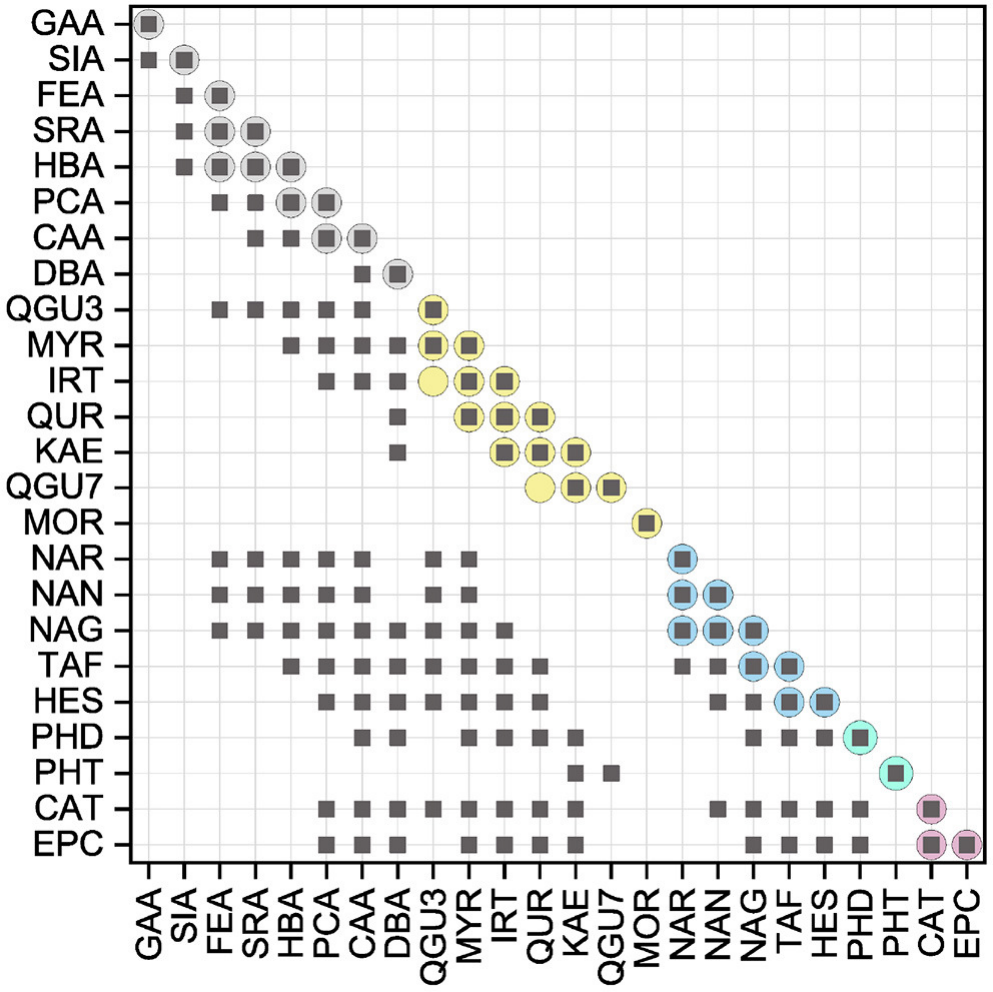
Results of the statistical analysis of the ORAC results with a significance level at *P* < 0.05. Squares indicate the results, which were not significantly different (*P* > 0.05) using all standard references. Circles indicate the results of the statistical analysis within the subgroups, which were not significantly different (*P* > 0.05).

The AUCs of all reference standards, divided into their subgroups, are shown in Figure 3. The results of the significance analysis using all reference standards are shown by squares in Figure 4. The ranking of reference standards was partly in agreement with previous studies (39, 46–49). In the following, the results of the individual subgroups are discussed, which is why the significance test was performed additionally within the respective group, as shown by circles in Figure 4.

When comparing the results of the phenolic acids, similar values were found, which did not differ significantly from each other. The presence of a catechol group had the strongest influence, which is why CAA and DBA achieved the highest values. PCA and HBA, which both have only one hydroxyl group, achieved lower values, which is consistent with literature (48, 50). The presence of a catechol group confers more stability to the B-ring by participating in electron delocalization thereby increasing the antioxidant activity (36, 43, 51–53). Due to resonance stabilization, *para*-*meta* hydroxylated substances exhibit enhanced antioxidant activity compared to monohydroxylated substances (50). However, if the molecule possesses a hydroxyl group in addition to the catechol group (galloyl group), this has a negative effect on the antioxidant activity in the ORAC assay, which is why GAA reached the lowest value and was partially in agreement with literature (54, 55). A lower value for a substance with a galloyl group compared to the catechol group was also shown in other studies, even if a different result was expected, due to the three hydroxyl groups on the aromatic ring (39, 54, 56–60). This can probably be attributed to steric hindrance.

The presence of additional methoxy groups to a hydroxyl group also leads to a lower value, which is why SIA, FEA and SRA achieved lower values than HBA and PCA. Again, steric hindrance is the propable cause for these results, just as with GAA, SIA, which also have three substituents, obtained a lower value than FEA. This has also been shown in other studies (39, 56–60). Furthermore, the replacement of a hydroxyl group by a methoxy group leads to a lower antioxidant activity, which can be explained by the fact that methylation leads to a decrease in active electron- and hydrogen-donating groups (50, 58, 61–63). In contrast to the hydroxycinnamic acids, the hydroxybenzoic acids have a higher antioxidant effect, which is why DBA and SRA achieved higher values than CAA and SIA. This result was expected to be different, because the additional conjugated double bond between the benzene ring and the carboxyl group leads to extended electron delocalization and should stabilize the resulting radical (50, 58, 61–63). HBA and PCA are exceptions here, as they do not differ significantly.

In contrast to the phenolic acids, the presence of a catechol group on the B-ring for the flavonols did not have the highest influence on the result. In their case, the substitution of the B-ring plays a minor role, contrary to what might be assumed from the first Bors criterion. KAE with only one hydroxyl group on the B-ring did not obtain a significantly different result as QUR, which has a catechol group. This result was expected to be different, since the B-ring of flavonoids is considered to be the most reactive group and the delocalization of the unpaired electron preserves the stability of the phenoxyl radical. Moreover, an intramolecular hydrogen bond is formed by a catechol group, which should further enhance the π-delocalization in the B-ring (38, 43, 58, 64–66). MOR, which has two hydroxyl groups, but not a catechol group, obtained the highest result in this study. A possible explanation could be the rotation of the B-ring leading to a stabilization of the O-radical at C-2' by a intramolecular hydrogen bond with 3-OH (42, 67). MYR, which has a galloyl group on the B-ring, showed a lower value in comparison to KAE with only one hydroxyl group and no significant different result when compared to QUR. We assume that steric hindrance of the molecule may occur if the substituents are adjacent to each other, leading to a reduction of the antioxidant behavior of these compounds in the ORAC assay. In addition to hydroxyl groups, C/O-glycosides and O-methylation also have a negative influence on the results, as shown by some studies (39, 56–60). There was also a negative effect with IRT, KAE, and QUR, although it should be noted that an additional hydroxyl group instead of O-methylation does not improve the antioxidant behavior either (QUR=IRT). The work of Kang et al. (68) also showed lower values for substances that have a methoxy group instead of a hydroxyl group on the benzene ring. Different results were also obtained for O-glycosylation. Thus, no significant difference was obtained for the substances QGU7 and QUR. Therefore, the presence of a sugar residue at C-7 seems not to have any influence on the result. This does not apply for QGU3 and QUR, where the sugar at C-3 has a prooxidative effect. A hydroxyl group at C-3 supports the reaction with the free radical, which is further favored by the presence of a hydroxyl group at C-5 and C-7 by electron donating effect (69, 70). The presence of Bors 2 and a 3-OH group on the C-ring also affected the antioxidant behavior in previous studies (39, 71). The presence of both structural properties additionally benefits the electron delocalization increasing the antioxidant activity (58, 64–66).

Also the presence of a galloyl group had no influence on the result, which is why MYR and QUR also did not achieve a significantly different value, which is not consistent with literature (72). MOR, having two hydroxyl groups, obtained a higher value than KAE with only one hydroxyl group and QUR with a catechol group. This result is consistent with the studies of Promden et al. (73), who also showed that the presence of a hydroxyl group at 3' and 5' strongly increases the result in the ORAC. The presence of a hydroxyl group in *ortho* position results in stabilization of the phenoxyl radical due to intramolecular hydrogen bonding leading to increased antioxidant activity (58, 74, 75). The results are also partially in agreement with those of Žuvela et al. (37) and not in agreement with Cao et al. (47). Again, KAE showed a higher value than MYR and QUR, which was in agreement with the work of Ishimoto et al. (49). Thus, two hydroxyl groups on the B-ring seem to improve the result as long as they are not adjacent to each other. Furthermore, the result of the ORAC assay was not negatively influenced by a methoxy group at C-3' of the B-ring instead of a hydroxyl group, leading to similar results for IRT and QUR, which was also shown in literature (49). The influence of the second Bors criterion cannot be assessed on the basis of our measurements, since it is fulfilled by all substances used. The only substance that does not fulfill the third Bors criterion (QGU3) achieved the lowest value. Therefore, the third Bors criterion seems to be the decisive one. Additionally, a sugar residue in place of a hydroxyl group at C-7 of the A-ring had no influence on the AUC, which is why QGU7 and QUR achieved similar values.

For the flavanones, just as for the flavonols, the presence of a hydroxyl group at C-7 of the A-ring had no effect on the antioxidant activity, which is why NAN and NAG, as well as NAN and NAR did not show a significant difference in AUC. In contrast to the phenolic acids and the flavonols, substances with two substituents on the B-ring obtained the highest values. However, it does not seem to matter whether this is a hydroxyl or a methoxy group, which is why TAF and HES achieved similar results and HES had a higher value than NAN. One possible explanation is the increase of the electron accessibility of the carbon atoms in the aromatic ring leading to a high antioxidant behavior for electron donating groups such as a methoxy group in *para* position (76, 77). The influence of a hydroxyl group at C-3 of the C-ring is not clear from these results, since only TAF has this group. Since TAF achieved a higher value than NAN, it is possible that there is a positive influence. However, since the structures differ in other features, these could have an additional influence. Since HES reached a higher value than NAN, a hydroxyl group in *para*-position does not have any influence on the result in the ORAC assay, although this property is considered to promote an antioxidant behavior (11, 13, 14, 78, 79). The influence of a hydroxyl group at C-3 of the C-ring is also not clear from our results.

The results of the dihydrochalcones were significantly different from each other, with PHT obtaining a higher value than PHD. Thus, a hydroxyl group at C-6' of the A-ring instead of a sugar residue had a positive influence on the result.

There was no significant difference for the flavanols, which was expected since CAT and EPC are structural isomers and was also shown in literature (49).

### 3.3. Comparison of the ORAC Assay With the DPPH, ABTS and FC

**Figure 5:**
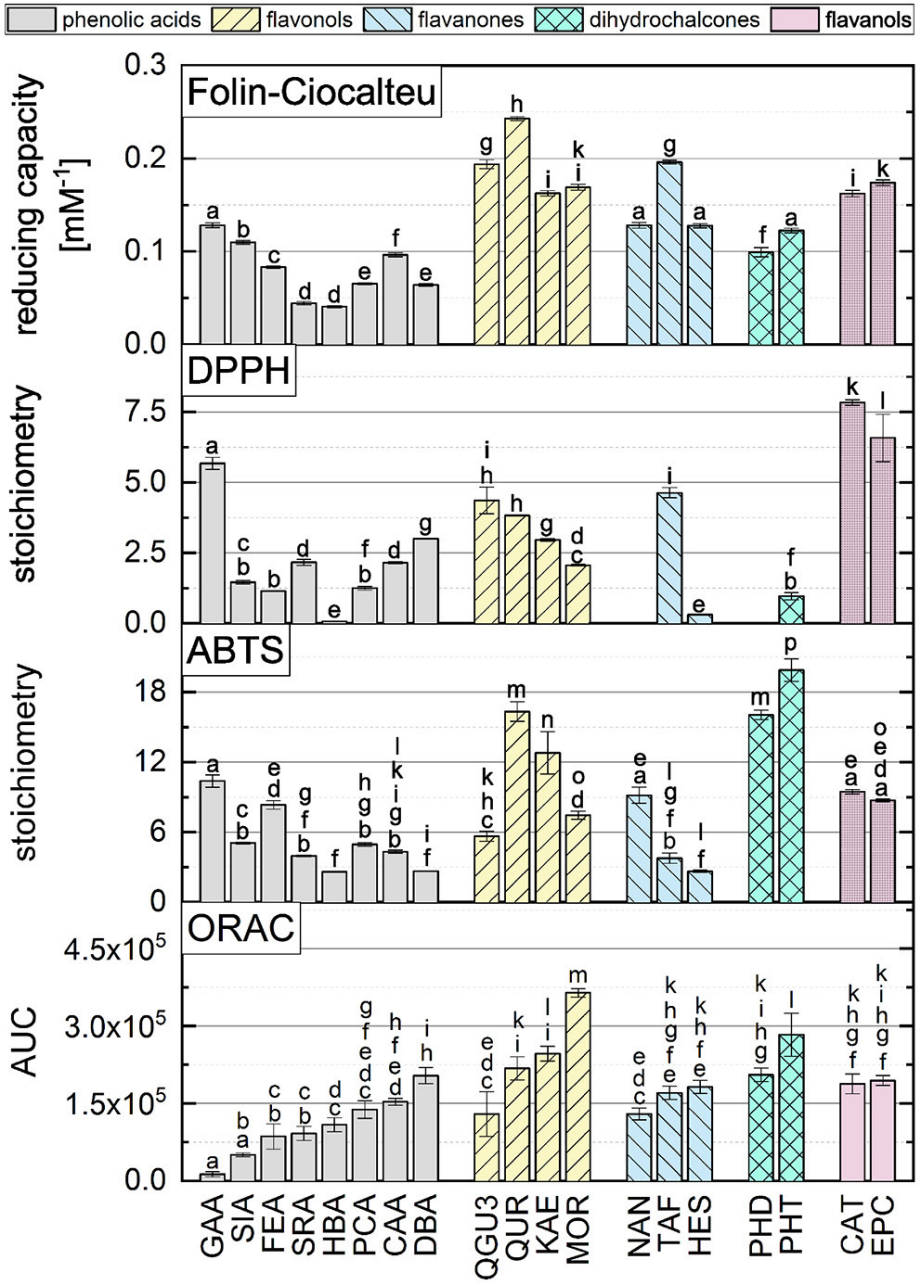
The AUC of all standard reference compounds measured in the ORAC assay in comparison to FC, DPPH and ABTS assays, which are reproduced from Platzer et al. (11, 14). Equal letters indicate that there is no significant difference between the results with an significance level at *P* > 0.05.

According to literature, there are different results on the correlation of different antioxidant *in vitro* methods (11, 14, 18, 19, 25–27). To investigate this in more detail, our ORAC assay results and structure activity relationships are compared to three SET-based assays (ABTS, DPPH and FC), which were published previously by Platzer et al. (11, 14). The results of the individual standard references are presented in Figure 5. In the following section, we discuss similarities and differences of the influences of structural properties leading to high values in the ABTS, DPPH, FC and ORAC assays.

No clear correlation can be found, when comparing the results of the four assays. This suggests that the assays are influenced by different structural properties. According to literature, the antioxidant behavior depends on both, number and position of hydroxyl groups and other substituents of a molecule (13).

For the group of phenolic acids, all assays showed higher values for the hydroxycinnamic acids than for the hydroxybenzoic acids, with exception of the DPPH assay. In the ABTS, DPPH and FC assays, in contrast to the ORAC assay, the results were not increased by an additional methoxy group. In the ABTS assay, substances with one hydroxyl and one methoxy group showed even higher values than substances with one catechol group. An increasing number of hydroxyl groups, also increased the results of a phenolic acid in the DPPH and FC assays. In the ABTS and ORAC assays, substances with a galloyl group showed significantly lower values, which could be explained by steric hindrance. In some cases, substances with one or two hydroxyl groups did not show significantly different values. For the DPPH and FC assays, the Bors criteria play a crucial role especially in the group of flavonols, with the first Bors criterion having the strongest influence. This effect was not seen for the ABTS or the ORAC assay. Substances with a galloyl group instead of a catechol group only achieved higher values in the DPPH. Again, steric hindrance may reduce the antioxidant behavior in the other assays. In all four assays, the third Bors criterion had an influence on the results, which is why QUR achieved higher values than QGU3. Substances which have an additional hydroxyl group in *ortho* position showed an increased value only in the ORAC assay. In the case of the flavanones, the second and the third Bors criterion had a positive influence on the results in the ABTS, DPPH and FC assays and also partially in the ORAC assay. A hydroxyl group in *para* position increased the results in all assays, with exception of the ORAC assay. The influence of a sugar residue in the four assays was either negative or there was no influence on the results. For the dihydrochalcones, PHT reached a higher value than PHD in all four assays, which can be explained by the additional hydroxyl group. PHD showed no reaction in the DPPH assay. For the two structural isomers EPC and CAT, which belong to the group of flavanols, similar values were reached in all four assays and it can be concluded that the three-dimensional arrangement of the molecules has no influence on the reaction in the *in vitro* methods (11, 14).

**Figure 6:**
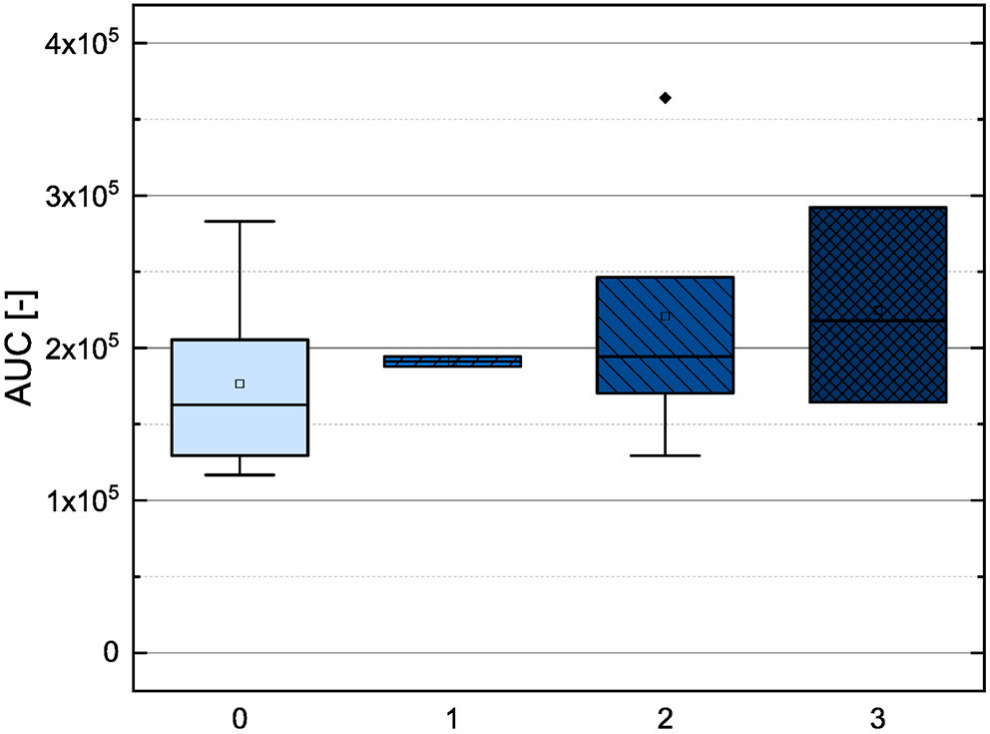
Boxplot of the mean values of results according to their number of fulfilled Bors criteria in the ORAC assay. The range within standard errors (1.5 interquartile range) is represented by error bars.

**Figure 7:**
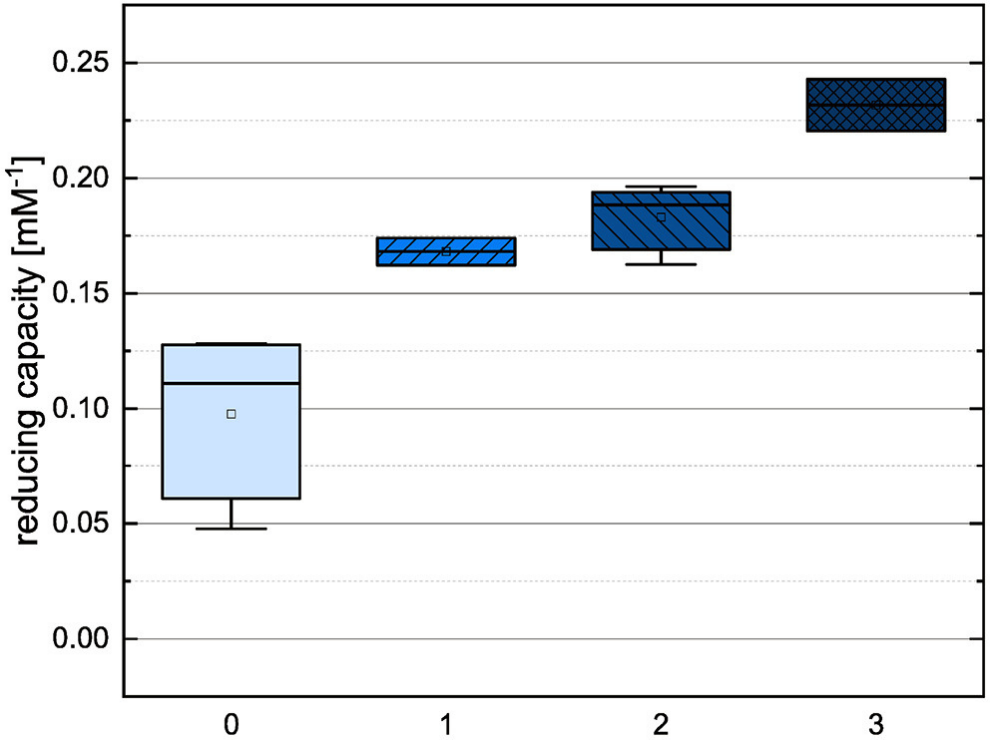
Boxplot of the mean values of results according to their number of fulfilled Bors criteria in the FC assay. The range within standard errors (1.5 interquartile range) is represented by error bars.

**Figure 8:**
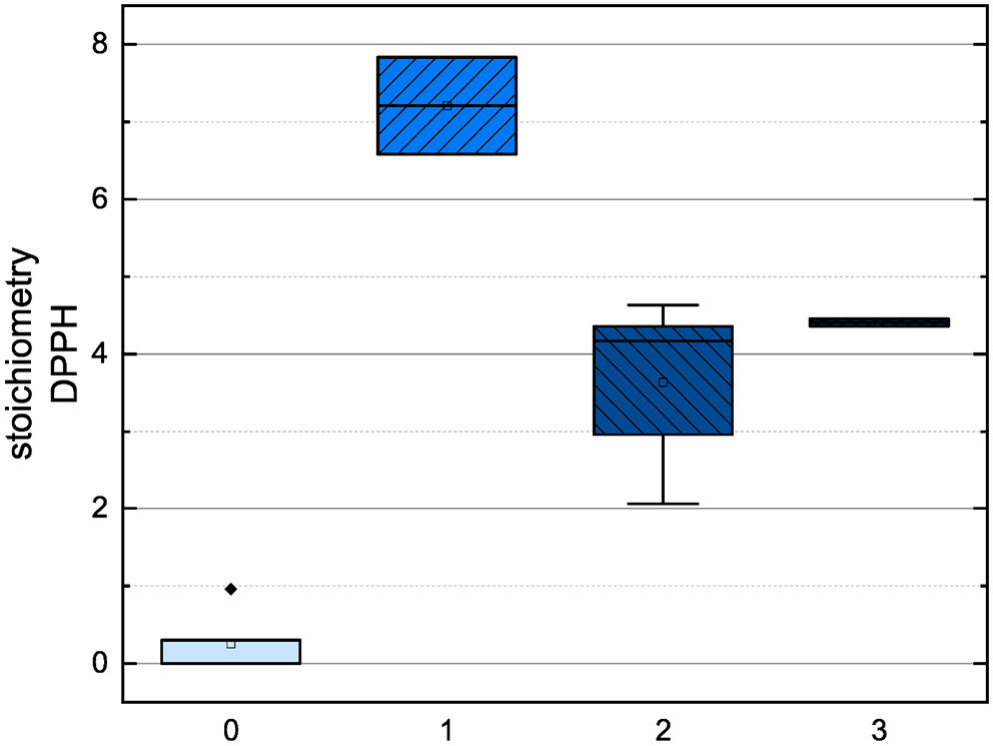
Boxplot of the mean values of results according to their number of fulfilled Bors criteria in the DPPH assay. The range within standard errors (1.5 interquartile range) is represented by error bars.

**Figure 9:**
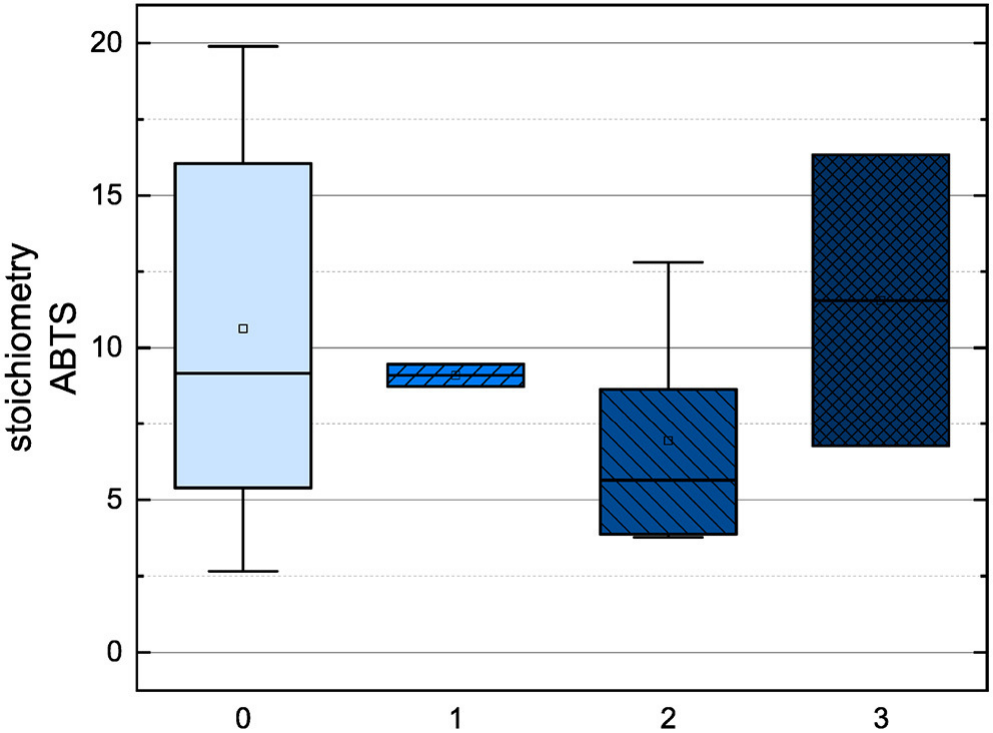
Boxplot of the mean values of results according to their number of fulfilled Bors criteria in the ABTS assay. The range within standard errors (1.5 interquartile range) is represented by error bars.

**Figure 10:**
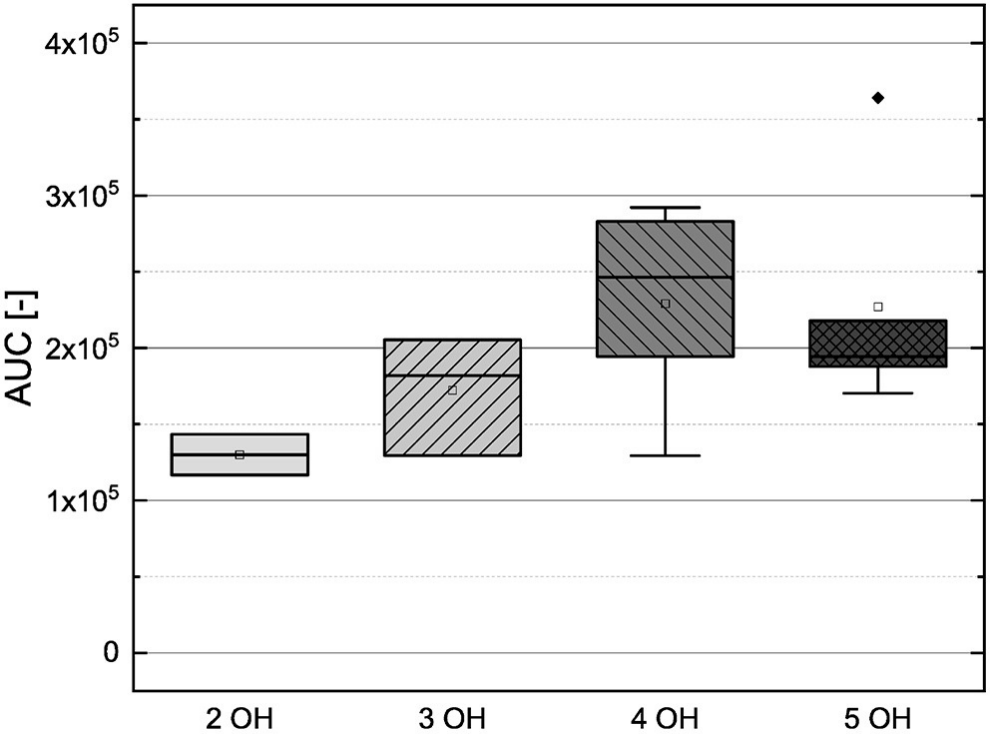
Boxplot of the mean values of results according to their number of hydroxyl groups in the ORAC assay. The range within standard errors (1.5 interquartile range) is represented by error bars.

**Figure 11:**
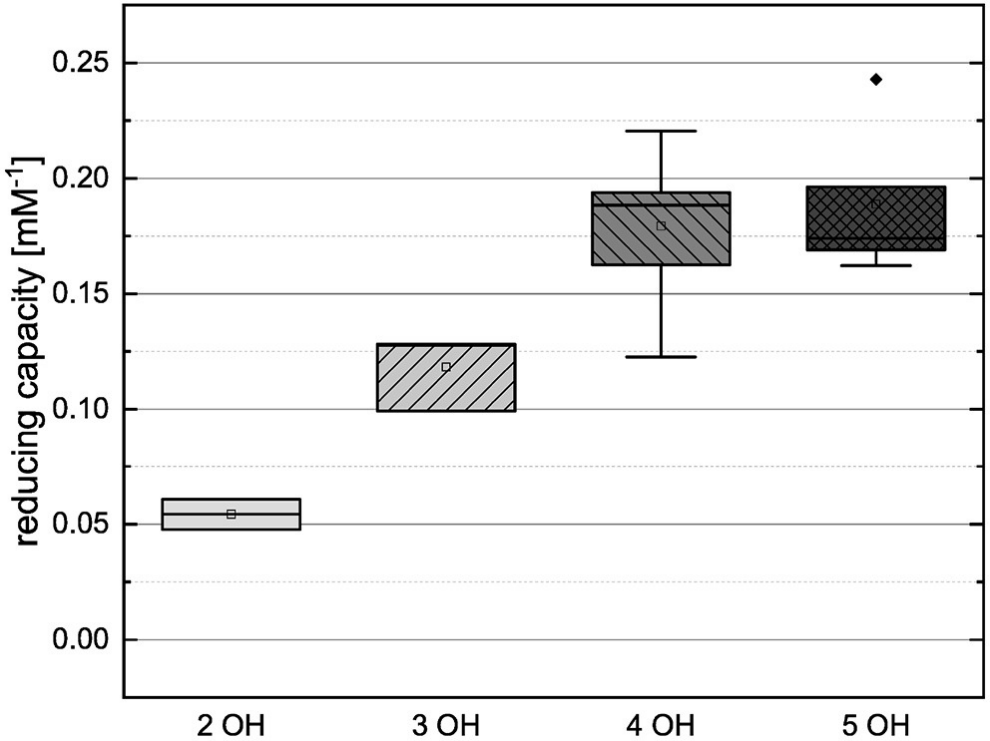
Boxplot of the mean values of results according to their number of hydroxyl groups in the FC assay. The range within standard errors (1.5 interquartile range) is represented by error bars.

**Figure 12:**
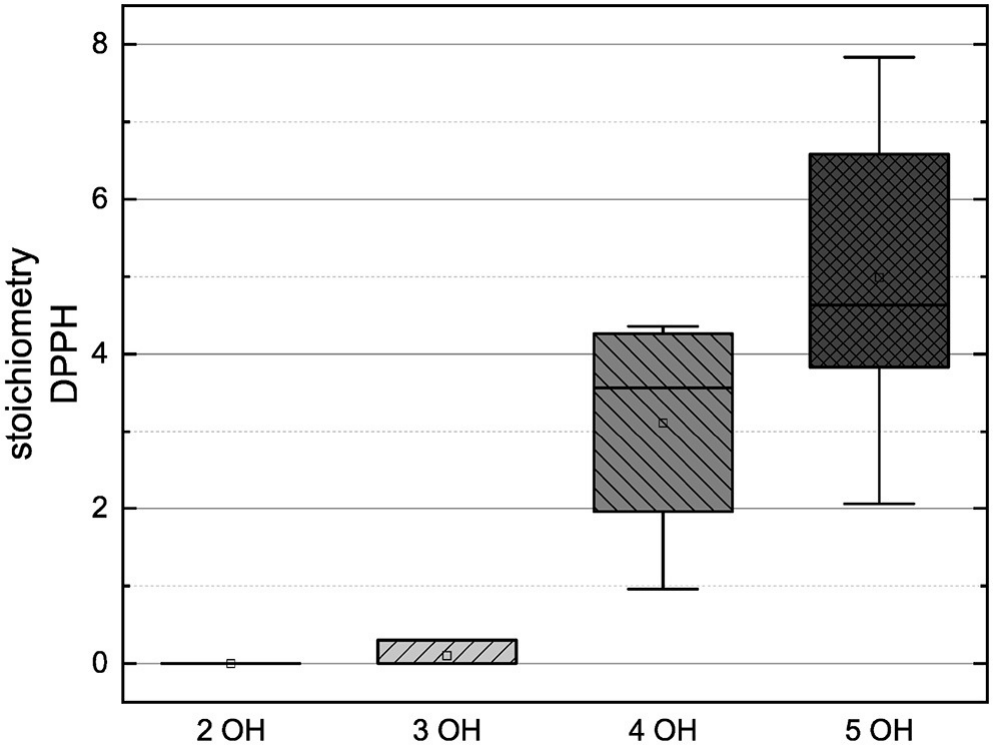
Boxplot of the mean values of results according to their number of hydroxyl groups in the DPPH assay. The range within standard errors (1.5 interquartile range) is represented by error bars.

**Figure 13:**
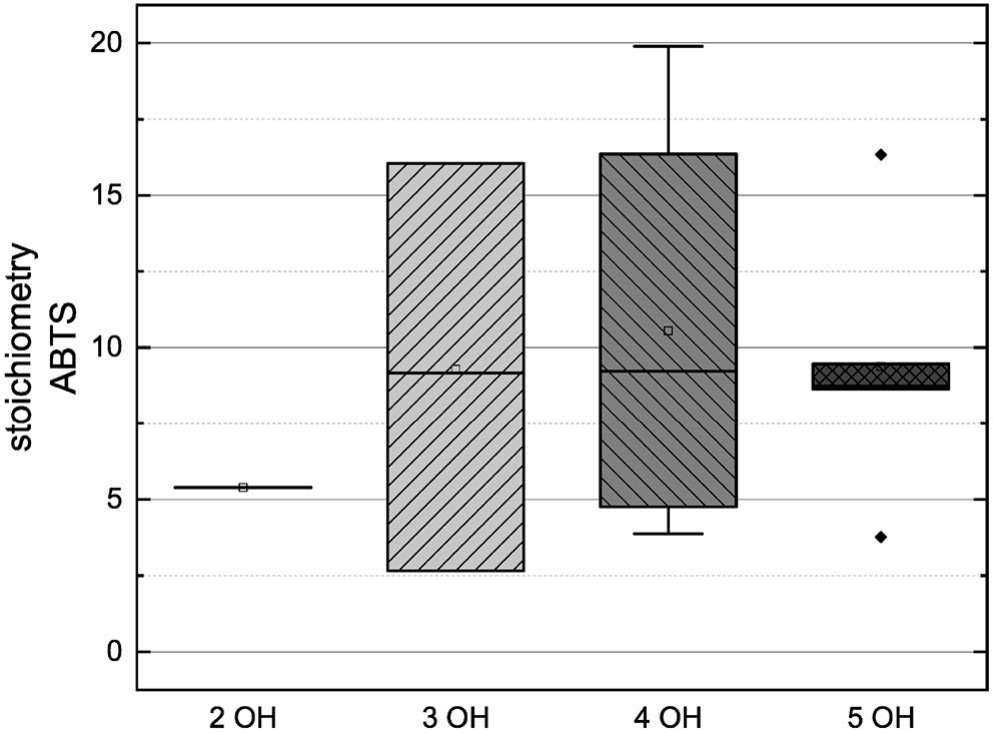
Boxplot of the mean values of results according to their number of hydroxyl groups in the ABTS assay. The range within standard errors (1.5 interquartile range) is represented by error bars.

In order to compare the influences of the Bors criteria and the number of hydroxyl groups on the results of the flavonoids in each assay (Figures 6-13) are presented. The number of Bors criteria had only a significant influence on the results of the FC assay. The results of the DPPH are not influenced by the number, but by the type of Bors criteria, with the first and the third Bors criterion having the strongest influence. The ABTS and the ORAC assays showed no clear correlation to the number or type of Bors criteria. In contrast to the ABTS assay, the results of the other assays appear to depend on the number of hydroxyl groups, with the values in the ORAC assay decreasing to some extent, if more than five hydroxyl groups are present (11, 14).

### 3.4. Principal Component Analysis of the DPPH, ABTS, FC and ORAC Results

**Figure 14:**
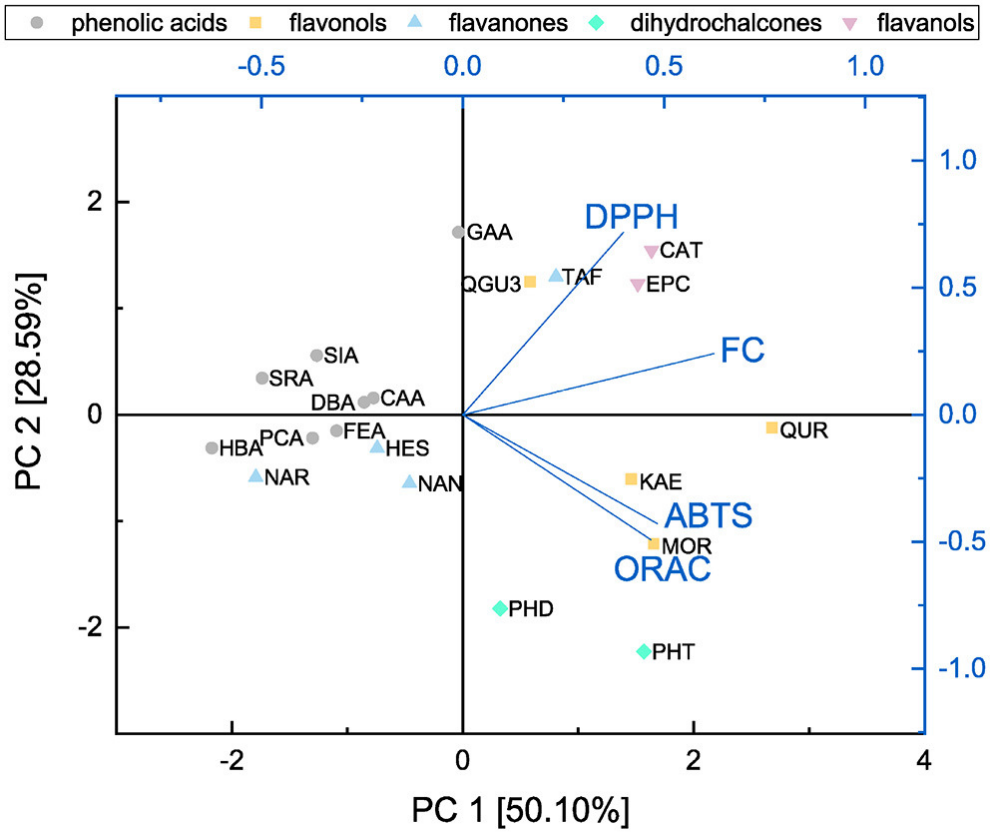
Principal component analysis of results from ABTS, DPPH, FC, and ORAC assays presented as a function of subgroups.

**Figure 15:**
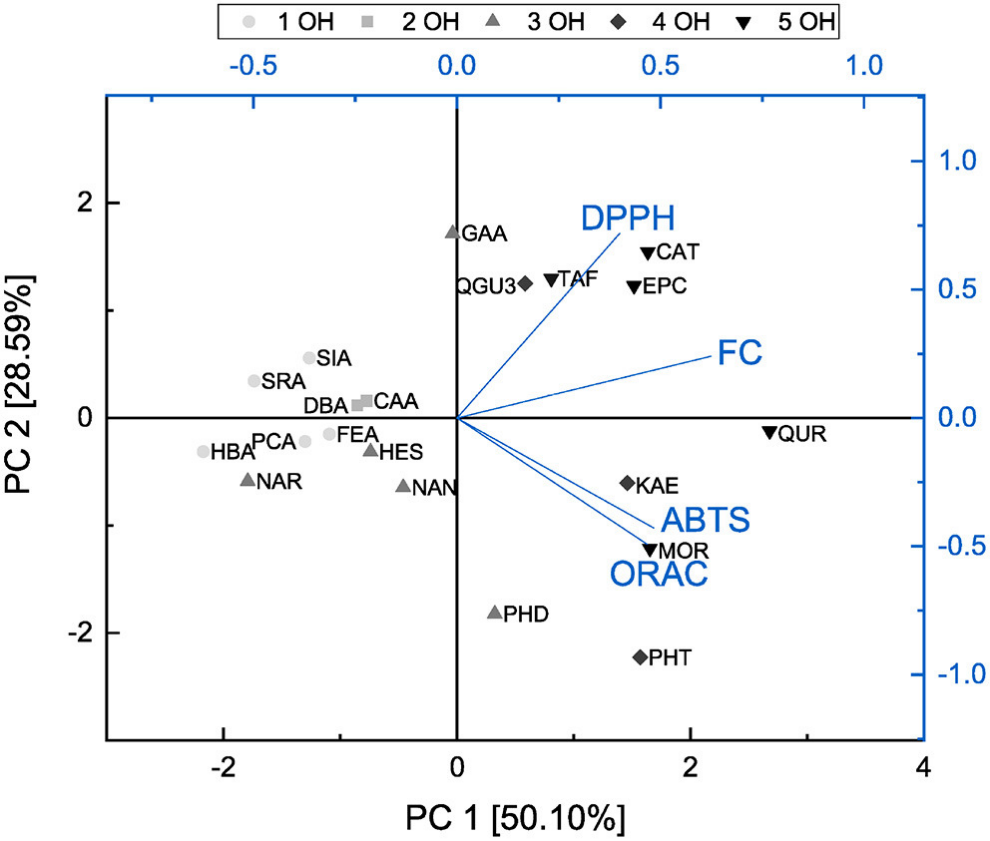
Principal component analysis of results from ABTS, DPPH, FC, and ORAC assays presented as a function of number of hydroxyl groups.

In order to identify the possible correlations between the assays and analyze structural properties relationship in more detail, a principal component analysis was performed and the results are shown in Figures 14, 15.

Both principal components cover 78.69 % of the initial variance of the original dataset. Principal component 1 represents the antioxidant behavior and depends on the results of all four assays. The higher the loading value of principal component 1 was, the higher the antioxidant effect was considering all four assays. The loading value of the FC assay is mostly correlated with principal component 1. This might be caused by affecting all subgroups equally well and therefore, we conclude that the FC assay is the most suitable to determine the antioxidant effect of the measured substances as previously reported (11). Furthermore, the FC and DPPH assays proceed in opposite direction to the ABTS and ORAC assays regarding principal component 2 and accordingly are influenced by different structural properties. The loading plots of the FC and DPPH assays point in the same direction, which can be explained as they follow the same reaction mechanism, i.e., SET. Since the loading plots of the ABTS and ORAC assays are very similar, it is possible that they follow the same reaction mechanism in our study. Also in the literature it is often not clear to which reaction mechanisms the ORAC and the ABTS can be assigned, since this depends above all also on the polarity of the solvent used (30, 31, 37, 38, 80, 81).

The score values of the different antioxidants show a clustering according their subgroups in the principal component analysis. Compared with the loading values of the four different assays the ABTS and ORAC seem to be particularly sensitive to the group of dihydrochalcones and rather to detect the substances of the flavanones and flavanols subgroups. For their determination, the FC and DPPH assays seem to be best suited. The substances also arrange with increasing number of hydroxyl groups along principal component 1 and therefore, this structural feature has a strong influence on the antioxidant behavior. With an increasing number of hydroxyl groups, the antioxidant effect increased. The influence of the fulfilled Bors criteria was also analyzed but no clear trend was shown. Nevertheless, an additional Bors criterion could have a positive influence, since for example QUR achieved a higher value than MOR despite having the same number of hydroxyl groups, but different number of Bors criteria.

## 4. Conclusions

In order to investigate the structural influence of phenolic compounds on the outcome of the ORAC assay, we measured standard references belonging to five different subgroups, namely phenolc acids, flavonols, flavanones, dihydrochalcones, and flavanols. These results were compared to those from the ABTS, DPPH and FC assays, previously published by Platzer et al. (11, 14).

According to literature, the antioxidant behavior of the abovementioned substances is dominated by the substituents, whereas their backbone plays a minor role. This is also true for our results, where the results were similar for subgroups with different backbones. The number of hydroxyl groups present in the substances had the highest influence on the AUC. The more hydroxyl groups, the better the result, except for molecules with two or more hydroxyl groups next to each other. This may be due to steric hindrance. The influence of additional methoxy groups could not be clarified conclusively. A sugar residue at C-7 seems to have no influence on the result, whereas sugar residues at C-3 or C-5 reduced the antioxidant effect. Although, the importance of Bors criteria on the antioxidant behavior is often described in literature, their effect could not be clearly shown in our studies. Only the second Bors criterion showed a positive effect in the ORAC assay in some cases.

By comparison to the ABTS, DPPH and FC assays, no clear correlation was found and therefore, no general statements can be drawn for all assays. An increasing number of hydroxyl groups had a positive influence on the results of the DPPH, FC and ORAC assays, as long as no steric hinderance occurred. Hydroxyl in *para* position increased the results in the ABTS, DPPH and FC assays, but had a negative influence on the ORAC value. The same applied for a methoxy substitution. In all four assays a sugar residue had either no influence on the results or slightly reduced the antioxidant effect. The three-dimensional structure had no influence on any assay result. The DPPH and FC assays most likely correlated with the Bors criteria, in contrast to the ORAC and ABTS assays, where their effect was not found. Finally, the DPPH was the only assay where some substances did not react at all. The differences in the assays can be explained by the different reaction mechanisms and the use of different evaluation methods, solvents, pH values and model radicals.

Principal component analysis showed the dependence of all four assays on the antioxidant behavior. Furthermore, the FC and DPPH assays depended on different structural properties than the ABTS and ORAC assays and accordingly we assume that they follow different reaction mechanisms. In addition, the number of hydroxyl groups had a strong influence on the antioxidant activity, while an influence of the Bors criteria was only shown partially and in combination with the number of hydroxyl groups.

In summary, our studies revealed the influence of structural properties of substances belonging to the subgroups of phenolic acids, flavonols, flavanones, dihydrochalcones, and flavanols on their antioxidant activities. Little was known about the structural properties of standard references that have an impact on experimental results, especially for ORAC assay. The commonalities of SET- and HAT-based reaction mechanisms were mainly studied theoretically and they were only partly in agreement with our experimental findings. The principal component analysis additionally showed the suitability of the different assays for different subgroups and can be used in further studies to select the appropriate assays for the specific application. For example, the DPPH should be used for flavanol-rich samples, whereas the ABTS and ORAC assays are suitable for dihydrochalcones and non-glycosylated flavonols. The FC assay is the only assay that evaluates all subgroups equally and best represents total antioxidant activity.

## Data Availability Statement

The raw data supporting the conclusions of this article will be made available by the authors, without undue reservation.

## Author Contributions

MP and SK contributed to conception and design of the study. MP, SK, and TT contributed to methodology, data curation, and visualization. MP performed the statistical analysis and wrote the first draft of the manuscript. All authors contributed to manuscript revision, read, and approved the submitted version.

## Funding

This research was partly funded by the German Federal Ministry of Education and Research (Grant Nos. 031B0387A and 031B0360A).

## Conflict of Interest

The authors declare that the research was conducted in the absence of any commercial or financial relationships that could be construed as a potential conflict of interest.

## Publisher's Note

All claims expressed in this article are solely those of the authors and do not necessarily represent those of their affiliated organizations, or those of the publisher, the editors and the reviewers. Any product that may be evaluated in this article, or claim that may be made by its manufacturer, is not guaranteed or endorsed by the publisher.

## Abbreviations

AAPH, 2: 2'-azubis-(2'-methylpropionamidine) dihydrochloride
ABTS, 2: 2'-azino-bis (3-ethylbenzothiazoline-6-sulfonic acid)
AOH: antioxidant
AUC: area under the curve
CAA: caffeic acid
CAT: (+)-catechin
DBA: 3,4-dihydroxybenzoic acid
DPPH: 2,2-diphenyl-1-picrylhydrazyl
EPC: (−)-epicatechin
FC: Folin-Ciocalteu
FEA: ferulic acid
GAA: gallic acid
HAT: hydrogen atom transfer
HBA: 4-hydroxybenzoic acid
HES: hesperetin
IRT: isorhamnetin
KAE: kaempferol
MOR: morin
MYR: myricetin
NAG: naringin
NAN: naringenin
NAR: narirutin
ORAC: oxygen radical absorbance capacity
PCA: p-coumaric acid
PHD: phloridzin
PHT: phloretin
QGU3: quercetin-3-D-glucoside
QGU7: quercetin-7-glucoside
QUR: quercetin
SIA: sinapic acid
SET: single electron transfer
SRA: syringic acid
TAF: taxifolin.

## References

[1] Shadidi F Nazck M. Food Phenolic Sources Chemistry Effects Applications. Lancaster, PA: Pensylvania Technomic Publishing Company Co. (1995).

[2] Bennett RN Wallsgrove RM. Secondary metabolites in plant defence mechanisms. New Phytol. (1994) 127:617–33. 10.1111/j.1469-8137.1994.tb02968.x 33874382

[3] Crozier A Clifford MN Ashihara H. Plant Secondary Metabolites: Occurrence, Structure and Role in the Human Diet. John Wiley & Sons (2008).

[4] Shahidi F Zhong HJ Ambigaipalan P. Antioxidants: regulatory status. In: Bailey's Industrial oil and Fat Products. Hoboken (2005). p. 1–21.

[5] Zheng W Wang SY. Antioxidant activity and phenolic compounds in selected herbs. J Agric Food Chem. (2001) 49:5165–70. 10.1021/jf010697n 11714298

[6] Dragland S Senoo H Wake K Holte K Blomhoff R. Several culinary and medicinal herbs are important sources of dietary antioxidants. J Nutr. (2003) 133:1286–90. 10.1093/jn/133.5.1286 12730411

[7] Wang SY. Antioxidant capacity of berry crops and herbs. In: Oriental Food and Herbs - Chemistry and Health Effects. Washington D.C.: ACS Publications (2003).

[8] Wu X Beecher GR Holden JM Haytowitz DB Gebhardt SE Prior RL. Lipophilic and hydrophilic antioxidant capacities of common foods in the United States. J Agric Food Chem. (2004) 52:4026–37. 10.1021/jf049696w 15186133

[9] Strack D. Chapter 10: phenolic metabolism. Plant Biochem. (1997) 387–416. 10.1016/B978-012214674-9/50011-4

[10] D Archivio M Filesi C Di Benedetto R Gargiulo R Giovannini C Masella R. Polyphenols, dietary sources and bioavailability. Annali-Istituto Superiore di Sanita. (2007) 43:348. 18209268

[11] Platzer M Kiese S Herfellner T Schweiggert-Weisz U Eisner P. How does the phenol structure influence the results of the folin-ciocalteu assay? Antioxidants. (2021) 10:811. 10.3390/antiox10050811 34065207 PMC8160659

[12] Bors W Heller W Michel C Saran M. Radical chemistry of flavonoid antioxidants. In: Antioxidants in Therapy and Preventive Medicine. Boston: Springer (1990). p. 165–70. 10.1007/978-1-4684-5730-8_25 2244490

[13] Shahidi F Janitha P Wanasundara P. Phenolic antioxidants. Crit Rev Food Sci Nutrit. (1992) 32:67–103. 10.1080/10408399209527581 1290586

[14] Platzer M Kiese S Herfellner T Schweiggert-Weisz U Miesbauer O Eisner P. Common trends and differences in antioxidant activity analysis of phenolic substances using single electron transfer based assays. Molecules. (2021) 26:1244–61. 10.3390/molecules26051244 33669139 PMC7956415

[15] Prior RL Cao G. Analysis of botanicals and dietary supplements for antioxidant capacity: a review. J AOAC Int. (2000) 83:950–6. 10.1093/jaoac/83.4.950 10995120

[16] Re R Pellegrini N Proteggente A Pannala A Yang M Rice-Evans C. Antioxidant activity applying an improved ABTS radical cation decolorization assay. Free Radical Biol Med. (1999) 26:1231–7. 10.1016/S0891-5849(98)00315-3 10381194

[17] Sánchez-Moreno C. Methods used to evaluate the free radical scavenging activity in foods and biological systems. Food Sci Technol Int. (2002) 8:121–37. 10.1177/1082013202008003770

[18] Stevanato R Fabris S Momo F. New enzymatic method for the determination of total phenolic content in tea and wine. J Agric Food Chem. (2004) 52:6287–93. 10.1021/jf049898s 15453702

[19] Huang D Ou B Prior RL. The chemistry behind antioxidant capacity assays. J Agric Food Chem. (2005) 53:1841–56. 10.1021/jf030723c 15769103

[20] Lu L Qiang M Li F Zhang H Zhang S. Theoretical investigation on the antioxidative activity of anthocyanidins: a DFT/B3LYP study. Dyes Pigments. (2014) 103:175–82. 10.1016/j.dyepig.2013.12.015

[21] Vagánek A Rimarčík J Dropková K Lengyel J Klein E. Reaction enthalpies of OH bonds splitting-off in flavonoids: the role of non-polar and polar solvent. Comput Theor Chem. (2014) 1050:31–8. 10.1016/j.comptc.2014.10.020

[22] Xue Y Zheng Y An L Dou Y Liu Y. Density functional theory study of the structure-antioxidant activity of polyphenolic deoxybenzoins. Food Chem. (2014) 151:198–206. 10.1016/j.foodchem.2013.11.064 24423521

[23] Wang G Xue Y An L Zheng Y Dou Y Zhang L. Theoretical study on the structural and antioxidant properties of some recently synthesised 2, 4, 5-trimethoxy chalcones. Food Chem. (2015) 171:89–97. 10.1016/j.foodchem.2014.08.106 25308647

[24] Zheng YZ Deng G Liang Q Chen DF Guo R Lai RC. Antioxidant activity of quercetin and its glucosides from propolis: a theoretical study. Sci Rep. (2017) 7:1–11. 10.1038/s41598-017-08024-8 28790397 PMC5548903

[25] Dudonne S Vitrac X Coutiere P Woillez M Mérillon JM. Comparative study of antioxidant properties and total phenolic content of 30 plant extracts of industrial interest using DPPH, ABTS, FRAP, SOD, and ORAC assays. J Agric Food Chem. (2009) 57:1768–74. 10.1021/jf803011r 19199445

[26] Apak R Güçlü K Demirata B Özyürek M Çelik SE Bektaşoğlu B. Comparative evaluation of various total antioxidant capacity assays applied to phenolic compounds with the CUPRAC assay. Molecules. (2007) 12:1496–547. 10.3390/12071496 17909504 PMC6149428

[27] Granato D Shahidi F Wrolstad R Kilmartin P Melton LD Hidalgo FJ. Antioxidant activity, total phenolics and flavonoids contents: should we ban in vitro screening methods? Food Chem. (2018) 264:471–5. 10.1016/j.foodchem.2018.04.012 29853403

[28] Benzie IFF Strain JJ. Ferric reducing/antioxidant power assay: Direct measure of total antioxidant activity of biological fluids and modified version for simultaneous measurement of total antioxidant power and ascorbic acid concentration. In: Packer L editor. Oxidants and Antioxidants Part A. vol. 299 of Methods in Enzymology. 1st ed. London, UK: Elsevier-Academic Press; King's College London, UK (1999). p. 15–27. Available online at: https://www.sciencedirect.com/science/article/pii/S0076687999990055. 10.1016/s0076-6879(99)99005-5 9916193

[29] Singleton VL Rossi JA. Colorimetry of total phenolics with phosphomolybdic-phosphotungstic acid reagents. Am J Enol Vitic. (1965) 16:144–58.

[30] Prior RL Wu X Schaich K. Standardized methods for the determination of antioxidant capacity and phenolics in foods and dietary supplements. J Agric Food Chem. (2005) 53:4290–302. 10.1021/jf0502698 15884874

[31] Schaich KM Tian X Xie J. Hurdles and pitfalls in measuring antioxidant efficacy: a critical evaluation of ABTS, DPPH, and ORAC assays. J Funct Foods. (2015) 14:111–25. 10.1016/j.jff.2015.01.043

[32] Miller NJ Rice-Evans C Davies MJ Gopinathan V Milner A. A novel method for measuring antioxidant capacity and its application to monitoring the antioxidant status in premature neonates. Clin Sci. (1993) 84:407–12. 10.1042/cs0840407 8482045

[33] Whitehead T Thorpe G Maxwell S. Enhanced chemiluminescent assay for antioxidant capacity in biological fluids. Anal Chim Acta. (1992) 266:265–77. 10.1016/0003-2670(92)85052-8

[34] Gulcin I. Antioxidants and antioxidant methods: an updated overview. Arch Toxicol. (2020) 94:651–715. 10.1007/s00204-020-02689-3 32180036

[35] Cao G Alessio HM Cutler RG. Oxygen-radical absorbance capacity assay for antioxidants. Free Radical Biol Med. (1993) 14:303–11. 10.1016/0891-5849(93)90027-R 8458588

[36] Rasulev BF Abdullaev ND Syrov VN Leszczynski J. A quantitative structure-activity relationship (QSAR) study of the antioxidant activity of flavonoids. QSAR Combinat Sci. (2005) 24:1056–65. 10.1002/qsar.200430013

[37] Žuvela P David J Yang X Huang D Wong MW. Non-linear quantitative structure-activity relationships modelling, mechanistic study and in-silico design of flavonoids as potent antioxidants. Int J Mol Sci. (2019) 20:2328. 10.3390/ijms20092328 31083440 PMC6539043

[38] Zhang D Liu Y Chu L Wei Y Wang D Cai S. Relationship between the structures of flavonoids and oxygen radical absorbance capacity values: a quantum chemical analysis. J Phys Chem A. (2013) 117:1784–1794. 10.1021/jp307746c 23343226

[39] Zhang Q Yang W Liu J Liu H Lv Z Zhang C. Identification of six flavonoids as novel cellular antioxidants and their structure-activity relationship. Oxid Med Cell Longev. (2020) 2020:4150897. 10.1155/2020/4150897 33014269 PMC7525318

[40] Wright JS Johnson ER DiLabio GA. Predicting the activity of phenolic antioxidants: theoretical method, analysis of substituent effects, and application to major families of antioxidants. J Am Chem Soc. (2001) 123:1173–83. 10.1021/ja002455u 11456671

[41] Vagánek A Rimarčík J Lukeš V Klein E. On the energetics of homolytic and heterolytic OH bond cleavage in flavonoids. Computat Theor Chem. (2012) 991:192–200. 10.1016/j.comptc.2012.04.014

[42] Spiegel M Andruniów T Sroka Z. Flavones' and flavonols' antiradical structure-activity relationship—A quantum chemical study. Antioxidants. (2020) 9:461. 10.3390/antiox9060461 32471289 PMC7346117

[43] van Acker SA de Groot MJ van den Berg DJ Tromp MN Donné-Op den Kelder G van der Vijgh WJ. A quantum chemical explanation of the antioxidant activity of flavonoids. Chem Res Toxicol. (1996) 9:1305–1312. 10.1021/tx9600964 8951233

[44] Huang D Ou B Hampsch-Woodill M Flanagan JA Prior RL. High-throughput assay of oxygen radical absorbance capacity (ORAC) using a multichannel liquid handling system coupled with a microplate fluorescence reader in 96-well format. J Agric Food Chem. (2002) 50:4437–44. 10.1021/jf0201529 12137457

[45] Cao G Prior RL. Measurement of oxygen radical absorbance capacity in biological samples. Methods Enzymol. (1999) 299:50–62. 10.1016/S0076-6879(99)99008-0 9916196

[46] Aaby K Hvattum E Skrede G. Analysis of flavonoids and other phenolic compounds using high-performance liquid chromatography with coulometric array detection: relationship to antioxidant activity. J Agric Food Chem. (2004) 52:4595–603. 10.1021/jf0352879 15264888

[47] Cao G Sofic E Prior RL. Antioxidant and prooxidant behavior of flavonoids: structure-activity relationships. Free Radical Biol Med. (1997) 22:749–60. 10.1016/S0891-5849(96)00351-6 9119242

[48] Dávalos A Gómez-Cordovés C Bartolomé B. Extending applicability of the oxygen radical absorbance capacity (ORAC- fluorescein) assay. J Agric Food Chem. (2004) 52:48–54. 10.1021/jf0305231 14709012

[49] Ishimoto H Tai A Yoshimura M Amakura Y Yoshida T Hatano T. Antioxidative properties of functional polyphenols and their metabolites assessed by an ORAC assay. Biosci Biotechnol Biochem. (2012) 76:395–9. 10.1271/bbb.110717 22313776

[50] Spiegel M Kapusta K Kołodziejczyk W Saloni J Zbikowska B Hill GA. Antioxidant activity of selected phenolic acids-ferric reducing antioxidant power assay and QSAR analysis of the structural features. Molecules. (2020) 25:3088. 10.3390/molecules25133088 32645868 PMC7412039

[51] Burton GW Doba T Gabe E Hughes L Lee F Prasad L. Autoxidation of biological molecules. 4. Maximizing the antioxidant activity of phenols. J Am Chem Soc. (1985) 107:7053–65. 10.1021/ja00310a049

[52] Alov P Tsakovska I Pajeva I. Computational studies of free radical-scavenging properties of phenolic compounds. Curr Top Med Chem. (2015) 15:85–104. 10.2174/1568026615666141209143702 25547098 PMC4462847

[53] Foti MC. Antioxidant properties of phenols. J Pharm Pharmacol. (2007) 59:1673–85. 10.1211/jpp.59.12.0010 18053330

[54] Sakurai S Kawakami Y Kuroki M Gotoh H. Structure-antioxidant activity (Oxygen Radical Absorbance Capacity) relationships of phenolic compounds. Sci Rep. (2021) 10:2611. 10.21203/rs.3.rs-180961/v1

[55] Atala E Aspée A Speisky H Lissi E López-Alarcón C. Antioxidant capacity of phenolic compounds in acidic medium: a pyrogallol red-based ORAC (oxygen radical absorbance capacity) assay. J Food Composit Anal. (2013) 32:116–25. 10.1016/j.jfca.2013.09.007

[56] Van Acker SA Tromp MN Griffioen DH Van Bennekom WP Van Der Vijgh WJ Bast A. Structural aspects of antioxidant activity of flavonoids. Free Radical Biol Med. (1996) 20:331–42. 10.1016/0891-5849(95)02047-0 8720903

[57] Masuoka N Matsuda M Kubo I. Characterisation of the antioxidant activity of flavonoids. Food Chem. (2012) 131:541–5. 10.1016/j.foodchem.2011.09.02027707007

[58] Olszowy M. What is responsible for antioxidant properties of polyphenolic compounds from plants? Plant Physiol Biochem. (2019) 144:135–43. 10.1016/j.plaphy.2019.09.039 31563754

[59] Simić A Manojlović D Šegan D Todorović M. Electrochemical behavior and antioxidant and prooxidant activity of natural phenolics. Molecules. (2007) 12:2327–40. 10.3390/12102327 17978760 PMC6149165

[60] Li K Fan H Yin P Yang L Xue Q Li X. Structure-activity relationship of eight high content flavonoids analyzed with a preliminary assign-score method and their contribution to antioxidant ability of flavonoids-rich extract from *Scutellaria baicalensis* shoots. Arabian J Chem. (2018) 11:159–70. 10.1016/j.arabjc.2017.08.002

[61] Cuvelier ME Richard H Berset C. Comparison of the antioxidative activity of some acid-phenols: structure-activity relationship. Biosci Biotechnol Biochem. (1992) 56:324–5. 10.1271/bbb.56.324

[62] Piazzon A Vrhovsek U Masuero D Mattivi F Mandoj F Nardini M. Antioxidant activity of phenolic acids and their metabolites: synthesis and antioxidant properties of the sulfate derivatives of ferulic and caffeic acids and of the acyl glucuronide of ferulic acid. J Agric Food Chem. (2012) 60:12312–23. 10.1021/jf304076z 23157164

[63] Rice-Evans CA Miller NJ Paganga G. Structure-antioxidant activity relationships of flavonoids and phenolic acids. Free Radical Biol Med. (1996) 20:933–56. 10.1016/0891-5849(95)02227-9 8743980

[64] Freeman BL Eggett DL Parker TL. Synergistic and antagonistic interactions of phenolic compounds found in navel oranges. J Food Sci. (2010) 75:C570–6. 10.1111/j.1750-3841.2010.01717.x 20722912

[65] Pietta PG. Flavonoids as antioxidants. J Nat Prod. (2000) 63:1035–42. 10.1021/np9904509 10924197

[66] Vellosa JCR Regasini LO Khalil NM Bolzani VdS Khalil OA Manente FA. Antioxidant and cytotoxic studies for kaempferol, quercetin and isoquercitrin. Eclética Quimica. (2011) 36:07–20. 10.1590/S0100-46702011000200001

[67] Amić A Marković Z Marković JMD Stepanić V Lučić B Amić D. Towards an improved prediction of the free radical scavenging potency of flavonoids: the significance of double PCET mechanisms. Food Chem. (2014) 152:578–85. 10.1016/j.foodchem.2013.12.025 24444978

[68] Kang J Xie C Li Z Nagarajan S Schauss AG Wu T. Flavonoids from acai (Euterpe oleracea Mart.) pulp and their antioxidant and anti-inflammatory activities. Food Chem. (2011) 128:152–7. 10.1016/j.foodchem.2011.03.011 25214342

[69] Butković V Klasinc L Bors W. Kinetic study of flavonoid reactions with stable radicals. J Agric Food Chem. (2004) 52:2816–20. 10.1021/jf049880h 15137819

[70] Rezk BM Haenen GR van der Vijgh WJ Bast A. The antioxidant activity of phloretin: the disclosure of a new antioxidant pharmacophore in flavonoids. Biochem Biophys Res Commun. (2002) 295:9–13. 10.1016/S0006-291X(02)00618-6 12083758

[71] Wen L Zhao Y Jiang Y Yu L Zeng X Yang J. Identification of a flavonoid C-glycoside as potent antioxidant. Free Radical Biol Med. (2017) 110:92–101. 10.1016/j.freeradbiomed.2017.05.027 28587909

[72] Ronsisvalle S Panarello F Longhitano G Siciliano EA Montenegro L Panico A. Natural flavones and flavonols: relationships among antioxidant activity, glycation, and metalloproteinase inhibition. Cosmetics. (2020) 7:71. 10.3390/cosmetics7030071

[73] Promden W Monthakantirat O Umehara K Noguchi H De-Eknamkul W. Structure and antioxidant activity relationships of isoflavonoids from *Dalbergia parviflora*. Molecules. (2014) 19:2226–37. 10.3390/molecules19022226 24561331 PMC6271601

[74] Bendary E Francis R Ali H Sarwat M El Hady S. Antioxidant and structure-activity relationships (SARs) of some phenolic and anilines compounds. Ann Agric Sci. (2013) 58:173–81. 10.1016/j.aoas.2013.07.002

[75] Hoelz L Horta B Araújo J Albuquerque M de Alencastro RB Da Silva J. Quantitative structure-activity relationships of antioxidant phenolic compounds. J Chem Pharm Res. (2010) 2:291–306.

[76] Jiang Y Rakesh K Alharbi NS Vivek H Manukumar H Mohammed Y. Radical scavenging and anti-inflammatory activities of (hetero) arylethenesulfonyl fluorides: synthesis and structure-activity relationship (SAR) and QSAR studies. Bioorg Chem. (2019) 89:103015. 10.1016/j.bioorg.2019.103015 31158576

[77] Ghannay S Bakari S Ghabi A Kadri A Msaddek M Aouadi K. Stereoselective synthesis of enantiopure N-substituted pyrrolidin-2, 5-dione derivatives by 1, 3-dipolar cycloaddition and assessment of their in vitro antioxidant and antibacterial activities. Bioorganic Med Chem Lett. (2017) 27:2302–7. 10.1016/j.bmcl.2017.04.044 28434766

[78] Ma YT Cheung PC. Spectrophotometric determination of phenolic compounds by enzymatic and chemical methods a comparison of structure- activity relationship. J Agric Food Chem. (2007) 55:4222–8. 10.1021/jf070084w 17441728

[79] Arts MJ Dallinga JS Voss HP Haenen GR Bast A. A critical appraisal of the use of the antioxidant capacity (TEAC) assay in defining optimal antioxidant structures. Food Chem. (2003) 80:409–14. 10.1016/S0308-8146(02)00468-5

[80] Zulueta A Esteve MJ Frígola A. ORAC and TEAC assays comparison to measure the antioxidant capacity of food products. Food Chem. (2009) 114:310–6. 10.1016/j.foodchem.2008.09.033

[81] Litescu SC Eremia SA Tache A Vasilescu I Radu GL. The use of oxygen radical absorbance capacity (ORAC) and Trolox equivalent antioxidant capacity (TEAC) assays in the assessment of beverages' antioxidant properties. In: Preedy V editor. Processing and Impact on Antioxidants in Beverages. 1st ed. London, UK: Elsevier-Academic Press; King's College (2014). p. 245–51.

