# Supplementary material for: Radical Scavenging Mechanisms of Phenolic Compounds: A Quantitative Structure-Property Relationship (QSPR) Study
Source: Front Nutr. 2022 Apr 4;9:882458. doi: 10.3389/fnut.2022.882458 (PMC9013829; doi:10.3389/fnut.2022.882458)
Supplement: Supplementary file 1 [file Data_Sheet_1.pdf]

Supplementary Material

1 SUPPLEMENTARY DATA

Table S1. Raw data to Figure 5: The AUC of all standard reference compounds measured in the ORAC assay in comparison to FC, DPPH and ABTS assays, which are reproduced from Platzer et al. (11, 14).

Sample code	ORAC AUC [-]	ABTS stoichiometry [-]	DPPH stoichiometry [-]	FC reducing capacity [mM^{-1}]
GAA	$0.13 \times 10^5 \pm 0.05 \times 10^5$	10.38 ± 0.54	5.68 ± 0.21	0.13 ± 0.01
SIA	$0.51 \times 10^5 \pm 0.04 \times 10^5$	5.07 ± 0.08	1.46 ± 0.06	0.11 ± 0.01
FEA	$0.86 \times 10^5 \pm 0.24 \times 10^5$	8.34 ± 0.37	1.14 ± 0.01	0.08 ± 0.01
SRA	$0.92 \times 10^5 \pm 0.14 \times 10^5$	3.96 ± 0.08	2.17 ± 0.10	0.04 ± 0.01
HBA	$1.09 \times 10^5 \pm 0.13 \times 10^5$	2.61 ± 0.02	0.06 ± 0.01	0.04 ± 0.01
PCA	$1.38 \times 10^5 \pm 0.17 \times 10^5$	4.95 ± 0.16	1.25 ± 0.07	0.07 ± 0.01
CAA	$1.53 \times 10^5 \pm 0.07 \times 10^5$	4.31 ± 0.13	2.15 ± 0.03	0.10 ± 0.01
DBA	$2.04 \times 10^5 \pm 0.16 \times 10^5$	2.66 ± 0.04	3.01 ± 0.02	0.06 ± 0.01
QGU3	$1.29 \times 10^5 \pm 0.43 \times 10^5$	5.64 ± 0.43	4.36 ± 0.47	0.19 ± 0.01
QUR	$2.18 \times 10^5 \pm 0.23 \times 10^5$	16.33 ± 0.85	3.83 ± 0.02	0.24 ± 0.01
KAE	$2.46 \times 10^5 \pm 0.14 \times 10^5$	12.81 ± 1.82	2.96 ± 0.04	0.16 ± 0.01
MOR	$3.64 \times 10^5 \pm 0.08 \times 10^5$	7.45 ± 0.34	2.06 ± 0.03	0.17 ± 0.01
NAN	$1.29 \times 10^5 \pm 0.12 \times 10^5$	9.17 ± 0.69	0.00 ± 0.00	0.13 ± 0.01
TAF	$1.70 \times 10^5 \pm 0.13 \times 10^5$	3.76 ± 0.43	4.63 ± 0.19	0.20 ± 0.01
HES	$1.82 \times 10^5 \pm 0.13 \times 10^5$	2.66 ± 0.09	0.30 ± 0.01	0.13 ± 0.01
PHD	$2.05 \times 10^5 \pm 0.13 \times 10^5$	16.05 ± 0.40	0.00 ± 0.00	0.10 ± 0.01
PHT	$2.83 \times 10^5 \pm 0.42 \times 10^5$	19.90 ± 0.96	0.96 ± 0.13	0.12 ± 0.01
CAT	$1.88 \times 10^5 \pm 0.19 \times 10^5$	9.47 ± 0.18	7.84 ± 0.10	0.16 ± 0.01
EPC	$1.94 \times 10^5 \pm 0.09 \times 10^5$	8.74 ± 0.12	6.58 ± 0.84	0.17 ± 0.01
